# Shear wave elastography as a reliable tool in the prediction of renal histopathological abnormalities

**DOI:** 10.1186/s12880-025-02137-7

**Published:** 2026-01-24

**Authors:** Hend Gamal Abu El Fadl, Mohammed K. Nassar, Rasha Shemies, Ahmed E. Abdulgalil, Mohamed Abdalbary, Fatma E. H. Moustafa, Doaa Khedr Mohamed Khedr

**Affiliations:** 1 Department of Diagnostic and Interventional Radiology, Faculty of Medicine, Mansoura University, Mansoura, Egypt; 2 Mansoura Nephrology and Dialysis Unit, Mansoura University, Mansoura, Egypt; 3 Department of Pathology, Mansoura University, Mansoura, Egypt

**Keywords:** Lupus nephritis, Chronic kidney disease, Supersonic shear imaging, Renal elastography, Interstitial fibrosis

## Abstract

**Background:**

Chronic kidney disease (CKD) is a global health burden with irreversible progression and cardiovascular risk. Renal biopsy, while the gold standard for assessing kidney pathology, is invasive. Supersonic shear imaging (SSI) elastography is a promising non-invasive tool for quantifying tissue stiffness, potentially reflecting underlying fibrosis. This study aimed to assess the diagnostic value of SSI elastography for predicting renal histopathological abnormalities based on tissue stiffness and to evaluate its correlation with biopsy findings.

**Methods:**

This cross-sectional study included 51 adult patients undergoing native kidney biopsy at Mansoura University Hospitals. All patients underwent SSI elastography before biopsy. Renal stiffness was measured in kilopascals in both kidneys. Histopathological findings, including interstitial fibrosis, tubular atrophy, and glomerulosclerosis, were correlated with elastographic data.

**Results:**

Median right and left kidney stiffness were 7 (5.6–8.5) and 7.7 (6.8–10) kPa, respectively. Right kidney stiffness showed significant correlations with age (*r* = -0.28, *p* = 0.04) and eGFR (*r* = 0.288, *p* = 0.04). In non-lupus nephritis cases, right kidney stiffness correlated negatively with serum creatinine (*r* = -0.409, *p* = 0.02) and interstitial fibrosis (*r* = -0.377, *p* = 0.03), and positively with eGFR (*r* = 0.438, *p* = 0.01). ROC analysis yielded an AUC of 0.706 (*p* = 0.05) for predicting eGFR < 30 mL/min/1.73 m², with a cutoff of 6.9 kPa (sensitivity 71%, specificity 79%).

**Conclusions:**

Supersonic shear wave elastography (SWE) is a promising non-invasive modality for predicting renal histopathological changes and assessing kidney function in CKD patients.

**Supplementary Information:**

The online version contains supplementary material available at 10.1186/s12880-025-02137-7.

## Introduction

Chronic kidney disease (CKD) represents a growing global health concern, affecting an estimated 10–15% of the population and exhibiting a progressive, irreversible nature that often leads to end-stage kidney disease (ESKD) [1, 2]. Beyond renal dysfunction, CKD significantly increases cardiovascular morbidity and mortality, further complicating disease management [3]. Early detection of CKD is paramount to delaying its progression and preventing associated complications. Among the key pathological drivers of CKD are glomerulosclerosis, interstitial fibrosis, tubular atrophy, and chronic inflammation—changes that are ultimately confirmed through histopathological examination of renal tissue [4].

Despite being the gold standard for evaluating renal histopathology, kidney biopsy remains an invasive procedure with potential risks, including bleeding, arteriovenous fistulas, and, rarely, leading to nephrectomy [5, 6]. While non-invasive parameters such as estimated glomerular filtration rate (eGFR) and proteinuria are commonly used to monitor CKD, they may not fully reflect the extent of underlying histological damage and are often insensitive to early fibrotic changes [7]. Therefore, developing reliable non-invasive techniques to assess renal pathology is crucial.

Recent advances in imaging, including ultrasound-based elastography and magnetic resonance elastography, have provided new opportunities for non-invasive fibrosis assessment [8, 9]. Among these, Supersonic Shear Imaging (SSI)—a form of shear wave elastography—has shown promising results in evaluating tissue stiffness, a surrogate marker for fibrosis. Elastography quantifies tissue stiffness through Young’s modulus, measured in pascals or kilopascals, which correlates with interstitial fibrosis and overall histological damage [10, 11]. The SSI technique, due to its simultaneous acquisition of quantitative and color-coded elasticity maps, is particularly suited for assessing curved organs like the kidney. As such, investigating the utility of SSI in predicting renal histopathological abnormalities could support its use in routine clinical practice [12, 13].

This study aims to evaluate the diagnostic performance of SSI elastography in predicting renal histopathological abnormalities by assessing kidney tissue stiffness and its correlation with interstitial fibrosis and overall histological grading in patients undergoing native kidney biopsy.

## Patients and methods

### Study design and setting

This cross-sectional study was conducted at Mansoura University Hospitals between January 2020 and March 2024. This study included a total of 51 adult patients presenting with renal impairment and/or proteinuria, who were indicated for native kidney biopsy, and accepted to participate in the study alongside 20 healthy controls. All patients were recruited from the Nephrology and Dialysis Unit (both inpatient and outpatient clinics) of Mansoura University.

The study protocol was reviewed and approved by the Institutional Research Board (IRB) of Mansoura University (Approval Code: R.24.07.2697.R1). Written informed consent was obtained from all participants before enrollment. Confidentiality and privacy were strictly maintained, and all data collected was used solely for research purposes.

### Eligibility criteria

Inclusion criteria included patients aged 18 years or older with clinical evidence of kidney disease who were indicated for kidney biopsy. This included suspected lupus nephritis (guided by serology and active urinary findings), significant proteinuria (≥ 1 g/day or nephrotic range), proteinuria with renal impairment, isolated significant proteinuria, and unexplained renal impairment. Patients were required to be conscious and cooperative to complete the imaging procedure.

The control group consisted of 20 healthy individuals recruited from hospital staff and community volunteers who met specific inclusion criteria: absence of prior kidney disease, normal renal function tests (serum creatinine, eGFR, and urinalysis), and no existing comorbidities such as hypertension or diabetes. Exclusion criteria included kidney transplant recipients, patients receiving dialysis, and those with urinary tract obstruction. Patients deemed unfit for elastography, such as those with acute heart failure, altered level of consciousness, delirium, or dementia, were also excluded from the study.

## All the studied cases were subjected to the following

### Demographics and clinical data collection

For each patient, demographic data, including age, sex, hypertension status, diabetes mellitus status, smoking status, and disease duration (in months), were recorded. Clinical indications for biopsy (e.g., lupus nephritis, proteinuria, unexplained acute kidney injury) were documented.

### Laboratory assessment

Laboratory investigations were performed at the time of evaluation and included serum creatinine levels, 24-hour urinary protein excretion (expressed in mg/g creatinine), and urine analysis. Serological markers such as antinuclear antibody (ANA) and anti-double-stranded DNA (anti-dsDNA), along with complement levels (C3 and C4).

### Shear wave elastography technique

Initially, a conventional B-mode ultrasound examination was conducted, followed by color Doppler and shear wave elastography assessments utilizing an advanced SSI sono-elastography instrument (GE LOGIC E 9) outfitted with 4 MHz transducers for color duplex and SWE evaluations. Initially, standard ultrasound images were acquired to evaluate the anteroposterior dimension, parenchymal thickness, and echogenicity in relation to the perinephric fat of both kidneys.

The SWE examination is subsequently conducted on the B-mode ultrasound image, with the transducer maintained in a stable, pressure-free position perpendicular to the imaging plane for several seconds to minimize compression artifacts. The patient was instructed to retain their breath during this process. Kidney stiffness (KS) is quantitatively assessed; the average value is reported in kilopascals (kPa). Ten measurements of KS were obtained using a region of interest (ROI = 5 mm) centered on the kidney’s zone. The average KS value was determined as an indicator of fibrosis and expressed in kilopascals (kPa). SWE indicates that kidney rigidity values are subsequently correlated with color duplex and laboratory findings. The Tsukuba scoring system was employed to evaluate the elastographic color maps. The values acquired were documented before the kidney biopsy.

To minimize respiratory motion artifacts and improve measurement reproducibility, all SWE measurements were performed during a standardized breath-hold at deep inspiration. Patients were instructed to take a deep breath and hold it for a few seconds while the measurements were acquired. This standardized respiratory phase was applied consistently across all participants, as respiratory motion has been shown to influence tissue stiffness measurements in shear wave elastography [14].

### Renal biopsy and histopathological analysis

Elastography measurements were obtained within 24 h of a renal biopsy. Biopsy samples were evaluated at the Pathology Department of Mansoura University.

A kidney biopsy was performed based on standard clinical indications, according to KDIGO guidelines [15]. Indications included suspected lupus nephritis (guided by serology and active urinary findings), significant proteinuria (≥ 1 g/day or nephrotic range), proteinuria with renal impairment, unexplained renal impairment or rising serum creatinine, and unexplained acute kidney injury after excluding pre-renal and post-renal causes. In our cohort, these indications corresponded to lupus nephritis, proteinuria with renal impairment, isolated significant proteinuria, and unexplained AKI. All biopsies were performed on the left kidney as per routine clinical practice. Histopathological assessment included the number of glomeruli, percentage of glomerulosclerosis, degree of interstitial fibrosis, tubular atrophy, interstitial infiltration, interstitial edema, and vascular lesions. In cases of lupus nephritis, the ISN/RPS classification system was applied, and both activity and chronicity indices were calculated.

### Statistical methods

Statistical analysis was performed using SPSS version 20. Categorical variables were expressed as numbers and percentages, and continuous variables were reported as mean ± standard deviation for normally distributed data or median (interquartile range) for non-normally distributed data, based on the Shapiro–Wilk test. Comparisons between groups were conducted using appropriate tests (e.g., t-tests, Mann–Whitney U tests, chi-square tests). Correlation analysis was performed using Spearman’s rank correlation coefficient. Receiver Operating Characteristic (ROC) curve analysis was used to assess the diagnostic performance of elastographic measurements in predicting reduced renal function (eGFR < 30 mL/min/1.73 m²). Sensitivity, specificity, and area under the curve (AUC) were calculated. A p-value < 0.05 was considered statistically significant.

## Results

The study included 51 patients and 20 healthy controls. Baseline demographic and laboratory characteristics of patients compared to healthy controls are summarized in Table 1. Females predominated in the cohort, accounting for nearly 59%, while males accounted for about 41%. The studied patients had a mean BMI of approximately 34 (34.06 ± 11.7). Hypertension was present in a considerable proportion of patients (41.2%), whereas diabetes was less common, affecting about 12%. About 14% of the included patients were smokers. The median disease duration was 5 months, with an interquartile range of 2 to 12 months.

Laboratory evaluation revealed a median serum creatinine level of 2 mg/dL, ranging from 0.9 to 6 mg/dL. Median 24-hour urinary protein excretion was notably elevated at 2520 mg/g creatinine (IQR: 1587–4275), and the median urinary red blood cell count was 10 per high-power field (IQR: 4–15). Regarding serology, approximately 29% of patients were ANA-positive, 24% had anti-dsDNA antibodies, and about 10% had anti-HCV antibodies. Low complement levels were also noted, with C3 and C4 being reduced in 25.5% and 11.8% of patients, respectively.

**Table 1:** Demographic characteristics and laboratory data of the studied patients compared to controls

		Patient (51)	Control	*P*
**Age** *(Mean±SD)*		34.06±11.7	48.5±7.03	**<0.001**
**Gender ** *N (%)*	**Male**	4 (20)	0.093	0.093
	**Female**	16 (80)	16 (80)	
**BMI** *(kg/m*^*2*^*)*		27.13 ± 4.5	26.57 ± 4.06	0.625
**Hypertension** *N (%)*		21 (41.2)	0	NA
**Diabetes** *N (%)*		6 (11.8)	0	NA
**Smoking** *N (%)*		7 (13.7)	0	NA
**Disease duration (months):** *(Median – IQR)*		5 (2-12)	0	NA
**Serum Creatinine** *[mg/dL] (Median – IQR)*		2 (0.9-6)	0.88 (0.77-1.09)	**<0.001**
**24 hours Urinary Protein** *[mg/g creatinine(Median – IQR)*		2520 (1587-4275)	85 (65.25-103)	**0.001**
**Urinary RBCs** *[per HPF] (Median – IQR)*		10 (4-15)	NA	NA
**Positive Serology** *N (%)*	**ANA**	NA	NA	NA
	**Anti ds-DNA**	NA	NA	NA
	**Anti-HCV Ab**	NA	NA	NA
**Complement** *N (%)*	**Low C3**	NA	NA	NA
	**Low C4**	NA	NA	NA

Histopathological findings are detailed in Table 2. Lupus nephritis was the leading indication for kidney biopsy, accounting for 39.2% (*n* = 20) of patients reflecting the high referral pattern of SLE cases to our tertiary referral center. This was followed by proteinuria with renal impairment (23.5%, *n* = 12) and unexplained acute kidney injury (21.6%, *n* = 11). Isolated proteinuria prompted biopsy in 15.7% of patients (*n* = 8).

**Table 2:** Histopathological data

**No. of glomeruli** *(Median – IQR)*		15 (10-23)
**% of sclerotic glomeruli** *(Median – IQR)*		20 (0-72)
**Interstitial fibrosis** *N (%)*	**Negative**	13 (25.5)
	**0-25%**	10 (19.6)
	**25-50%**	10 (19.6)
	**> 50%**	18 (35.3)
**Tubular atrophy** *N (%)*	**Negative**	16 (31.4)
	**0-25%**	11 (21.6)
	**25-50%**	9 (17.6)
	**> 50%**	15 (29.4)
**Interstitial infiltration** *N (%)*	**Negative**	14 (27.5)
	**0-25%**	17 (33.3)
	**25-50%**	11 (21.6)
	**> 50%**	9 (17.6)
**Marked interstitial edema** *N (%)*		2 (3.9)
**Vascular lesions** *N (%)*	**No**	35 (68.6)
	**Thrombosis**	3 (5.9)
	**Hyalinosis**	1 (2)
	**Fibrointimal thickening**	12 (23.5)

Histopathological examination revealed evidence of lupus nephritis in 39% of the included cohort. Other abnormalities showed membranoproliferative glomerulonephritis (MPGN) (9.8%), followed by minimal change disease (5.9%), focal segmental glomerulosclerosis and membranous nephropathy (each 3.9%), and HCV-associated nephropathy (2%) in patients clinically presenting with nephrotic syndrome (25.5%). Less common abnormalities included vasculitis, thrombotic microangiopathy (TMA), and myeloma cast nephropathy, each constituting 2% of the sample. Nearly 29% of patients showed evidence of advanced chronic kidney disease or end-stage kidney disease (CKD/ESKD).

Histopathological analysis revealed a median glomerular count of 15 per biopsy sample (IQR: 10–23), with a range of 0%-72% sclerotic glomeruli (median: 20%). Interstitial fibrosis was present in over one-third (35.3%), demonstrating > 50% fibrosis. Similarly, nearly 30% of patients exhibited severe tubular atrophy (> 50%). Interstitial infiltration was common, with only 27.5% showing no infiltration, and 17.6% demonstrating extensive infiltration (> 50%). Marked interstitial oedema was infrequent, reported in only 3.9% of cases. Regarding vascular lesions, 68.6% of patients had no detectable abnormalities, while fibrointimal thickening was the most prevalent vascular finding (23.5%), followed by thrombosis (5.9%) and hyalinosis (2%).

Among patients diagnosed with lupus nephritis (LN), the mean age was approximately 31 years (31.05 ± 8.59). Females predominated the group, representing 85% of cases, while only 15% were males. One-quarter of the patients were hypertensive, whereas diabetes was absent, and only 5% had a history of smoking. The disease duration showed considerable variability, with a median of 12 months (IQR: 5.25–72).

Regarding laboratory results, LN patients had a median serum creatinine level of 1.25 mg/dL (IQR: 0.9–4.37) and a median 24-hour urinary protein excretion of 2400 mg/g creatinine (IQR: 1990–3700). Urinary red blood cell count was moderately elevated, with a median of 11.5 per high-power field (IQR: 5.25–30). Most patients were seropositive, with ANA detected in 70% and anti-dsDNA in 60%. None tested positive for anti-HCV antibodies. Complement levels were reduced in half of the patients for C3 (50%) and in 25% for C4.

Histopathological evaluation of LN patients revealed a median of 13.5 glomeruli per biopsy (IQR: 10–24). The majority of patients (60%) had class IV lupus nephritis, while class V and VI each accounted for 15%, and classes I and II were reported in 5% each; None of the investigated biopsies showed class III LN. Among the 12 patients with class IV LN, endocapillary hypercellularity was the most prominent active lesion, with a median score of 2.5, followed by cellular crescents (score 2), hyaline lesions (score 1.5), and neutrophilic infiltration (score 1). Fibrinoid necrosis was mainly absent. The median overall activity index was 8 (IQR: 7–10.75). Chronicity parameters revealed sclerotic glomeruli as the most common feature (median score 1), followed by atrophic tubules, fibrous crescents, and interstitial fibrosis—all with median scores around 1. One-third of patients showed 25–50% tubular atrophy or interstitial fibrosis, while 16.7% had > 50% involvement. The median chronicity index was 4 (IQR: 3–6).

Elastography results are summarized in Tables 3 and 4. Elastographic assessment of the total sample showed median stiffness values of 7 kPa (IQR: 5.6–8.5) in the right kidney and 7.7 kPa (IQR: 6.8–10) in the left kidney (Table 3). Elastographic findings in LN patients demonstrated higher stiffness in the left kidney (median: 8.65 kPa, IQR: 6.92–10.45) compared to the right kidney (median: 6.9 kPa, IQR: 4.97–8.65) (*P* = 0.01), indicating notable lateral differences in renal elasticity within this subgroup (Table 4). In contrast, no significant side-to-side differences were observed in patients with CKD/ESKD (*P* = 0.14) or those with nephrotic syndrome (*P* = 0.32).

**Table 3:** Elastographic findings (all patients)

	Group (*n* = 51)	Control (*n* = 20)	*p*
**Left kidney** *(Median – IQR)*	7.7 (6.8-10)	16.6 (13.6-24.2)	**<0.001**
**Right kidney** *(Median – IQR)*	7 (5.6-8.5)	16.4 (12.4-20.05)	**<0.001**

**Table 4:** Elastographic findings of LN patients (*n* = 20)

	LN (*n* = 20)	Non-LN (*n* = 31)	Control (*n* = 20)	*p*
**Left kidney** *(Median – IQR)*	8.65 (6.92-10.45)^c^	7.5 (6.6-9.1)^c^	16.6 (13.6-24.2)^a,b^	**<0.001**
**Right kidney** *(Median – IQR)*	6.9 (4.97-8.65)^c^	7 (5.6-8.1)^c^	16.4 (12.4-20.05)^a,b^	**<0.001**

Stratification of patient characteristics and shear wave speed by eGFR category is shown in Table 5. Patients were further compared according to eGFR values. Patients with preserved renal function (eGFR > 60 mL/min/1.73 m²) were significantly younger (mean age: 28.1 ± 7.6 years) than those with moderate (36.5 ± 7.1 years) or severely reduced eGFR (< 30 mL/min/1.73 m²; 37.9 ± 14 years), with *P* = 0.01. Although females predominated in the higher eGFR group (73.7%) and males were more common in the mid and low eGFR groups, this difference in gender distribution was not statistically significant (*P* = 0.23). Hypertension demonstrated a highly significant association with renal impairment, affecting only 5.3% of those with eGFR > 60 but rising sharply to 66.7% and 60.9% in those with eGFR 30–60 and < 30, respectively (*P* < 0.0001). No statistically significant differences were observed between the three eGFR groups in terms of 24-hour urinary protein excretion (*P* = 0.39). Likewise, although patients with higher eGFR had numerically higher renal stiffness values in both kidneys, differences in shear wave speed across groups did not reach statistical significance (right kidney: *P* = 0.2; left kidney: *P* = 0.28).

**Table 5:** Comparison of patient characteristics and shear wave speed based on eGFR categories

	eGFR > 60 (*n* = 19)	eGFR 30-60 (*n* = 9)	eGFR<30 (*n* = 23)	
Age	28.1±7.6^b,c^	36.5±7.1^a^	37.9±14^a^	0.01
Gender:				0.23
Male	5 (26.3)	5 (55.6)	11 (47.8)	
Female	14 (73.7)	4 (44.4)	12 (52.2)	
DM	0	1 (11.1)	5 (21.7)	0.09
HTN	1 (5.3)	6 (66.7)	14 (60.9)	<0.0001
24 hours urinary protein	3000 (1990-5300)	2160 (657-2902)	2500 (1450-4000)	0.39
Shear wave speed:				
Right kidney:	7.4 (6.5-8.7)	7 (6.3-9.8)	6.1 (5.5-7.6)	0.2
Left kidney:	8.8 (7-11.3)	7.8 (6.7-9.6)	7.3 (6.6-9.6)	0.28

Correlations between shear wave speed and clinical and histopathological variables are presented in Table 6. The shear wave speed of both kidneys was correlated with the studied variables. Right kidney shear wave speed revealed a significant negative correlation with age (*r* = − 0.28, *P* = 0.04) and a significant positive correlation with estimated glomerular filtration rate (eGFR) (*r* = 0.288, *P* = 0.04). In contrast, left kidney shear wave speed showed a borderline negative correlation with age (*r* = − 0.272, *P* = 0.053), and a non-significant correlation with eGFR (*P* = 0.53). No significant correlations were observed between shear wave speeds and serum creatinine (*P* = 0.06 and 0.07), urinary protein excretion (*P* = 0.28 and 0.5), percentage of sclerotic glomeruli (*P* = 0.33 and 0.47), interstitial infiltration (*P* = 0.45 and 0.2), interstitial fibrosis (*P* = 0.3 and 0.2), or tubular atrophy (*P* = 0.55 and 0.2) in either kidney.

**Table 6:** Correlations of shear wave speed of both kidneys with the studied variables (ALL cases: *n* = 51)

Parameter	Right kidney		Left kidney	
	*r*	*p*	*r*	*p*
Age	-0.28	0.04*	-0.272	0.053
Creatinine	-0.265	0.06	-0.255	0.07
eGFR	0.288	0.04*	0.273	0.53
protein	0.153	0.28	0.097	0.5
% of sclerotic glomeruli	-0.137	0.33	-0.1	0.47
Interstitial infiltration	-0.107	0.45	-0.18	0.2
Interstitial fibrosis	-0.148	0.3	-0.17	0.2
Tubular atrophy	-0.08	0.55	-0.15	0.2

**Fig. 1:**
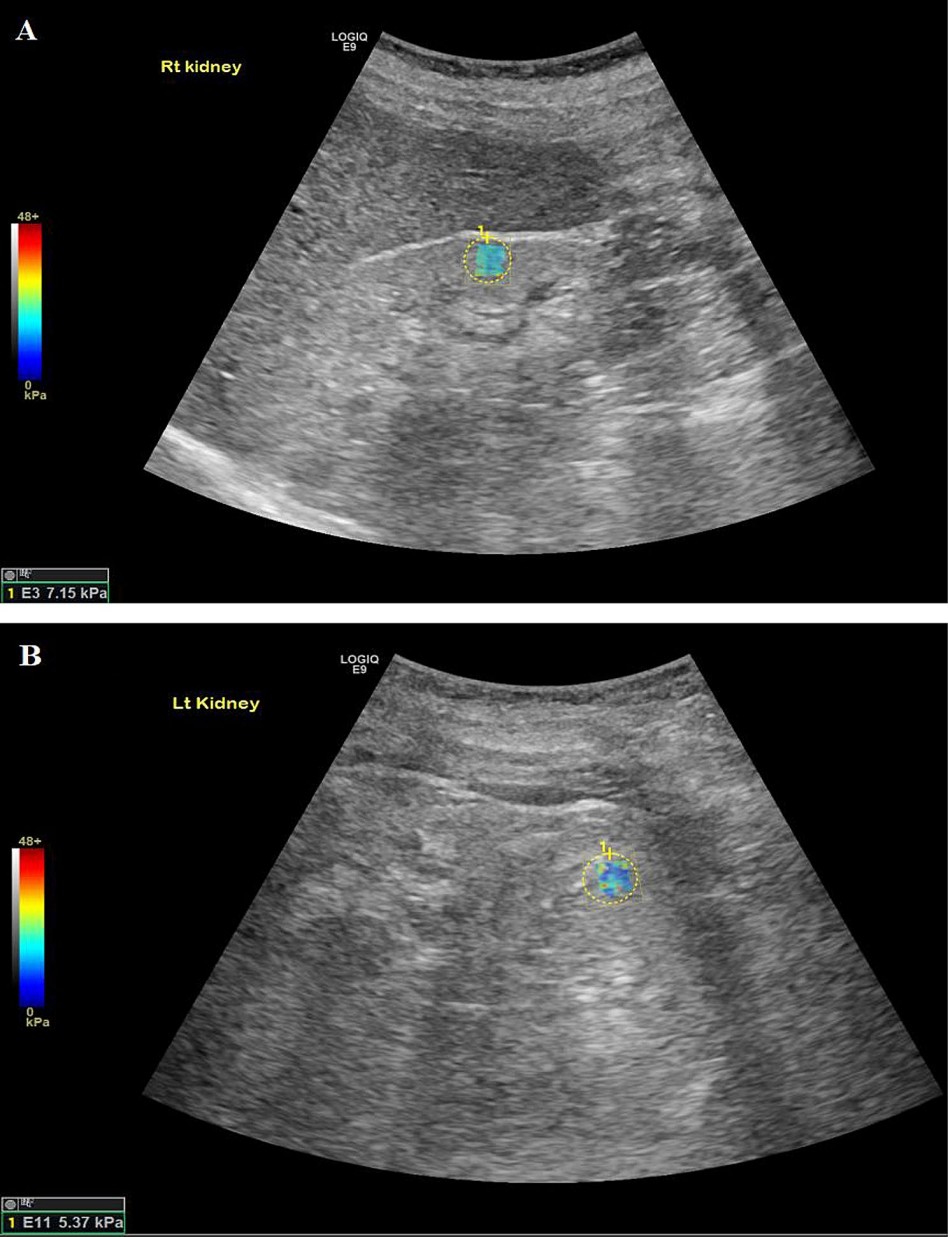
An 18-year-old patient with lupus nephritis class IV, chronicity index of 12/12 according to the International Society of Nephrology/Renal Pathology Society (ISN/RPS) criteria. The SSI of both kidneys reveal 7.15 kPa and 5.37 kPa of the right and left kidneys respectively

**Fig. 2:**
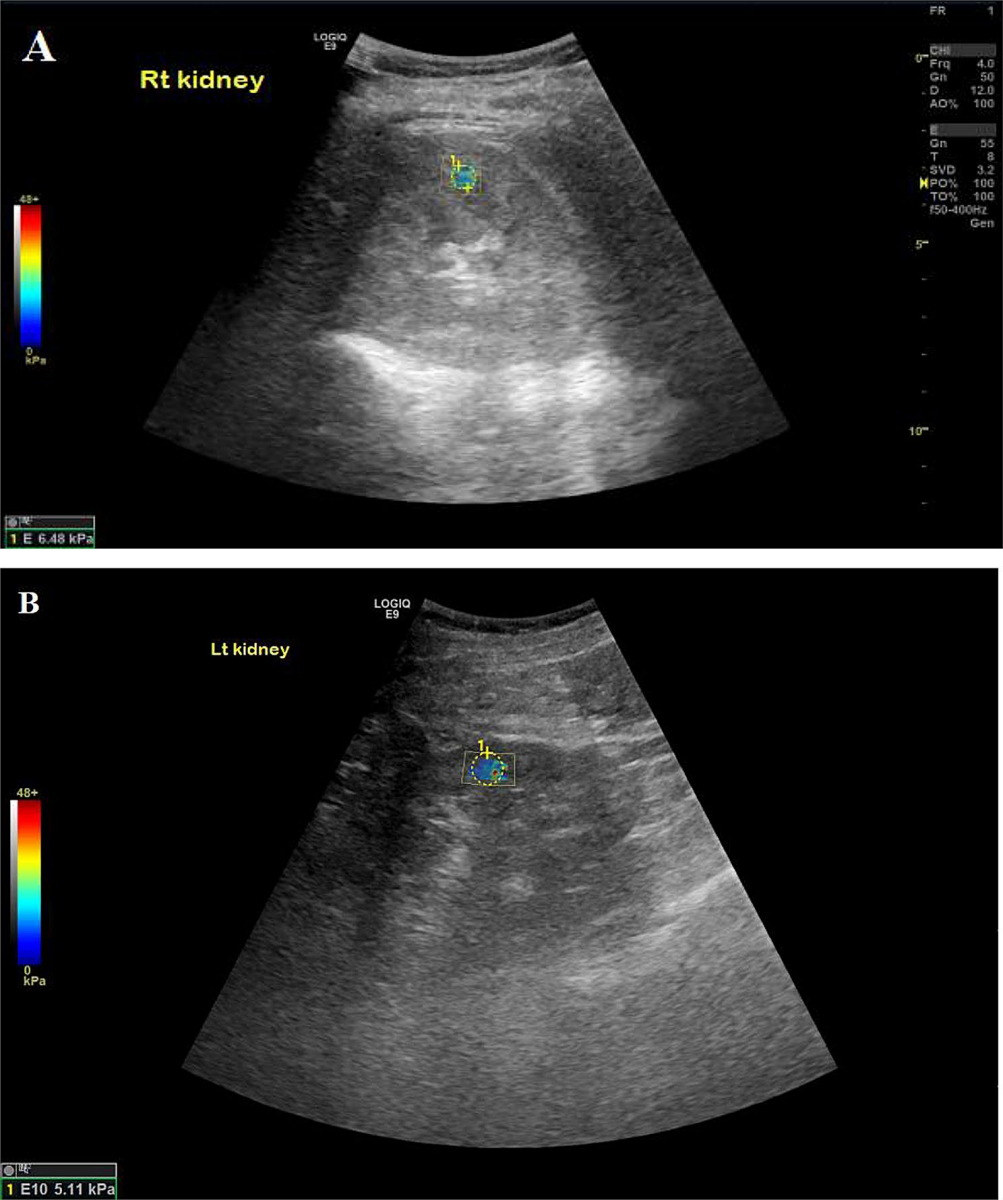
The SSI of two different patients with lupus nephritis reveal 6.48 kPa and 5.11 kPa of the right and left kidneys respectively. **A**, right kidney belong to a 17-year-old patient with lupus nephritis class IV, chronicity index 11; **B**, the left kidney belong to a 34-year-old patient with lupus nephritis class IV, chronicity index 5/12, both according to the International Society of Nephrology/Renal Pathology Society (ISN/RPS) criteria

**Fig. 3:**
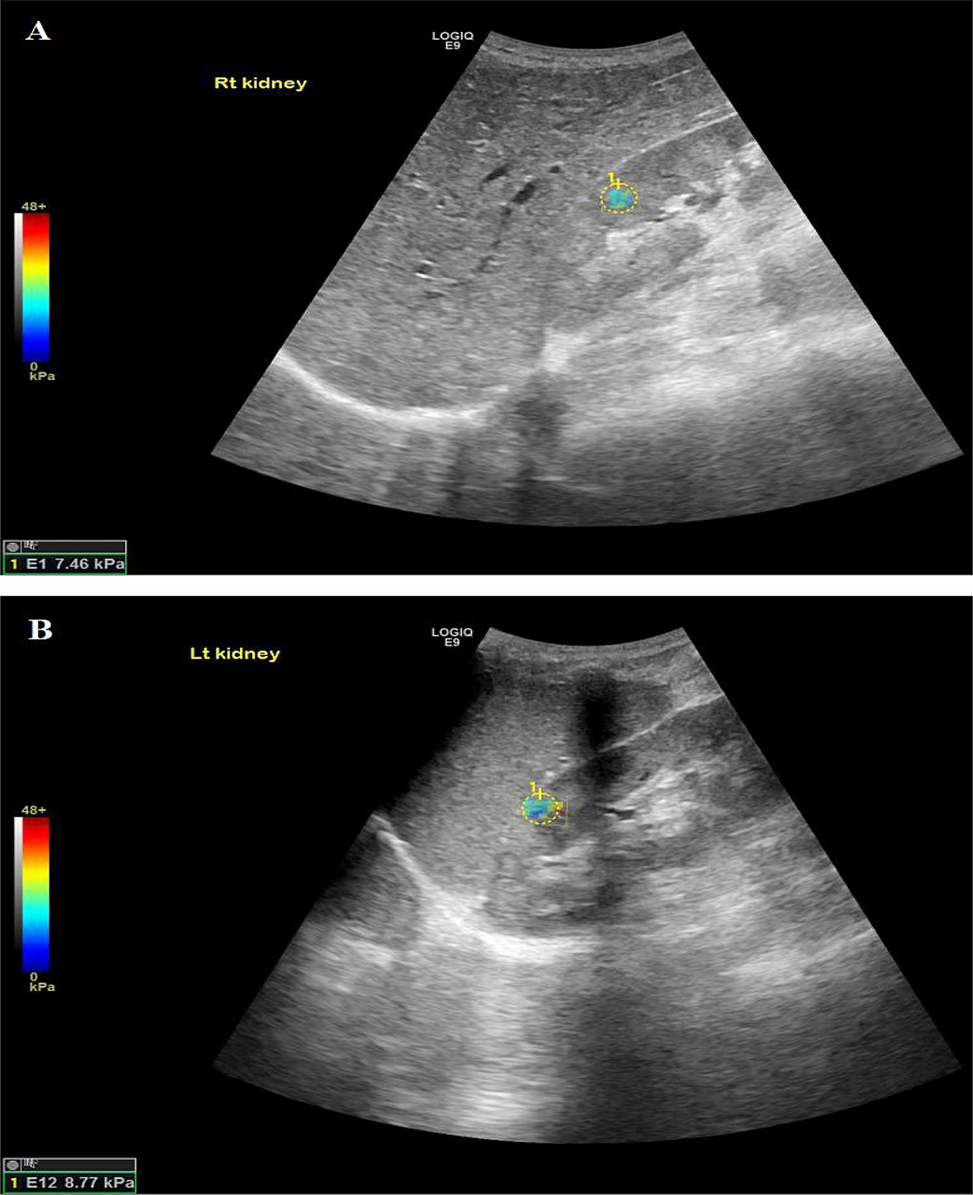
A 29-year-old patient with lupus nephritis class IV, chronicity index of 4/12 according to the International Society of Nephrology/Renal Pathology Society (ISN/RPS) criteria. The SSI of both kidneys reveal 7.46 kPa and 8.77 kPa of the right and left kidneys respectively

Among LN patients (Figs. 1, 2 and 3), left kidney shear wave speed revealed a significant positive correlation with neutrophilic infiltration/karyorrhexis score (*r* = 0.86, *P* = 0.007). No other significant correlations were observed between right or left kidney stiffness and clinical parameters, including age (*P* = 0.49 and 0.32), serum creatinine (*P* = 0.84 and 0.72), eGFR (*P* = 0.8 and 0.51), or proteinuria (*P* = 1 and 0.26). Similarly, there were no significant associations with histopathological activity components, including endocapillary hypercellularity, hyaline lesions, cellular crescents, fibrinoid necrosis, or interstitial infiltration (*P* > 0.1 for all). Additionally, no significant correlations were found between shear wave speed and chronicity components, including sclerotic glomeruli, fibrous crescents, tubular atrophy, interstitial fibrosis, or the overall chronicity index in either kidney (*P* > 0.1 for all).

In non-lupus nephritis (non-LN) cases, right kidney shear wave speed revealed significant negative correlations with serum creatinine (*r* = − 0.409, *P* = 0.02) and interstitial fibrosis (*r* = − 0.377, *P* = 0.03), and a significant positive correlation with eGFR (*r* = 0.438, *P* = 0.01). No significant correlations were found between right kidney stiffness and age (*P* = 0.06), proteinuria (*P* = 0.25), percentage of sclerotic glomeruli (*P* = 0.07), interstitial infiltration (*P* = 0.13), or tubular atrophy (*P* = 0.15). Similarly, left kidney stiffness did not show significant correlations with any of the assessed clinical or histopathological variables, including age, creatinine, eGFR, proteinuria, sclerotic glomeruli, interstitial infiltration, interstitial fibrosis, or tubular atrophy (*P* > 0.05 for all).

**Fig. 4:**
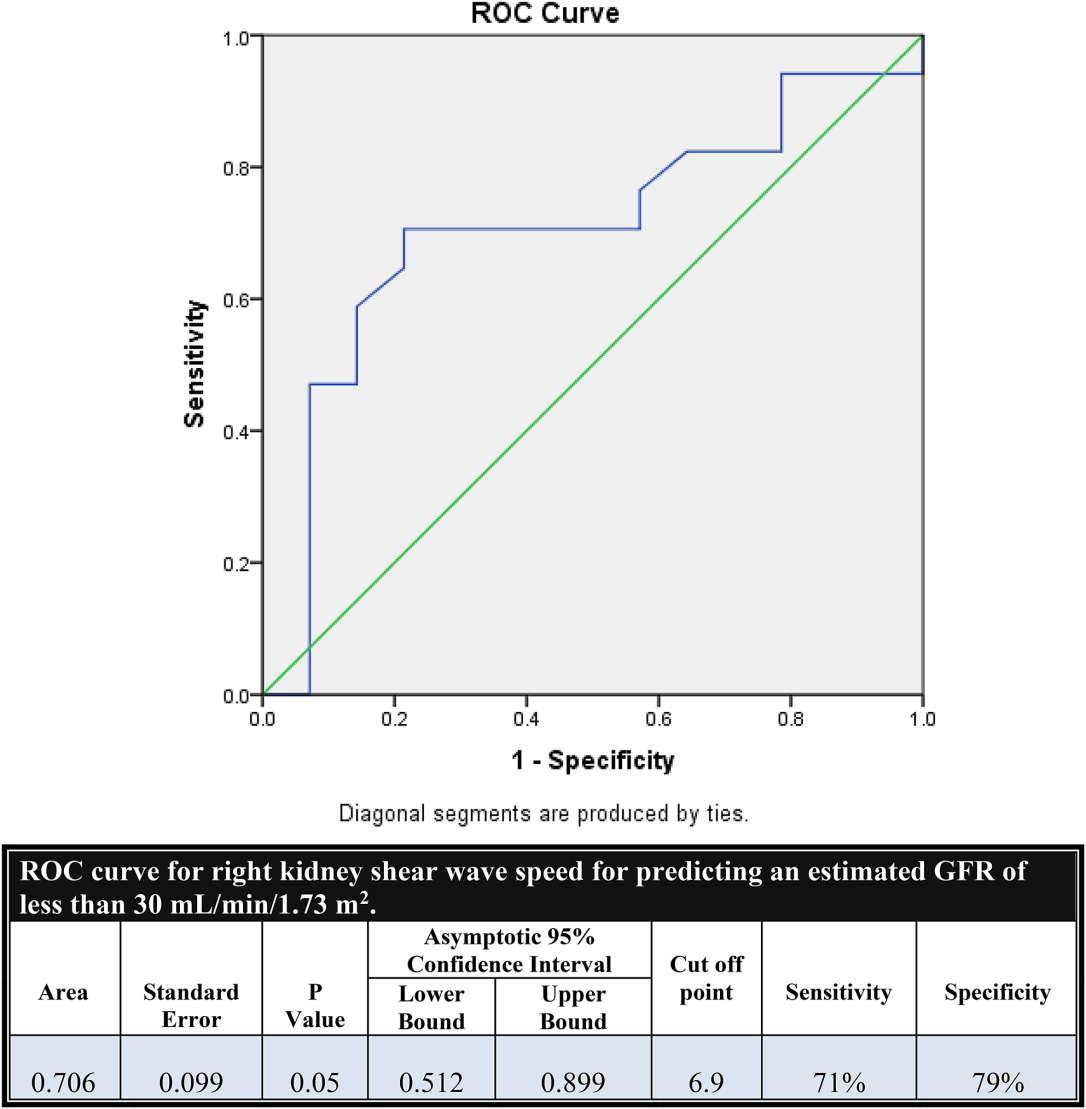
ROC curve of Right kidney shear wave speed for prediction of eGFR <30 in non-LN cases

ROC curve analysis was conducted for right kidney shear wave speed to predict an estimated GFR of less than 30 mL/min/1.73 m² in non-lupus nephritis cases. It showed a significant AUC of 0.706 (*P* = 0.05), with a 95% confidence interval of 0.512 to 0.899, indicating good ability to discriminate advanced renal dysfunction. The optimal cutoff value was 6.9 kPa, which yielded a sensitivity of 71% and a specificity of 79% (Fig. 4).

## Discussion

Optimum management of CKD requires early detection, but the gold standard tool used for diagnosis of kidney disease, the kidney biopsy, is invasive and carries a risk of complications. Supersonic SWE offers a promising, non-invasive alternative for assessing renal stiffness [16, 17]. Therefore, this study aims to evaluate the diagnostic value of shear wave elastography in predicting renal histopathological abnormalities, particularly interstitial fibrosis, and the overall histological score in patients with native kidney disease.

The present study compared the shear wave speed of both kidneys in patients with biopsy-proven kidney diseases (mainly due to LN) versus healthy controls. Although left kidney biopsy provides the standard for diagnosis of systemic diseases, its findings may not perfectly represent the exact degree of damage in the right kidney which may imply a different level of fibrosis or structural change in that kidney. Furthermore, the right kidney often provides a better acoustic window for reliable Shear Wave Elastography (SWE) measurements due to the liver interface which often results in more reliable SWE measurements compared to the left kidney, where bowel gas and spleen can interfere. This led the present study to consider both kidneys in the assessment.

Shear wave elastography revealed significantly reduced renal cortical stiffness in both kidneys of patients with lupus nephritis compared to healthy controls (*p* < 0.001). These findings indicate that renal involvement in lupus nephritis is associated with a measurable decline in cortical stiffness, supporting elastography as a helpful non-invasive tool for detecting parenchymal alterations.

As CKD progresses, reduced renal blood flow, loss of functioning nephrons, and decreased parenchymal turgor may override the stiffening effect of patchy interstitial fibrosis, yielding lower shear-wave speeds. In accordance with this, Bota S et al. [18] observed significantly lower shear wave velocities in CKD patients than in controls, supporting SWE as a marker of parenchymal damage. Also, Lakshmana et al. [19] found significantly lower cortical stiffness (Young’s modulus) in CKD patients, with a moderate negative correlation with eGFR. A pilot study by Samir et al. [20] included 25 CKD patients (median GFR 38) vs. 20 healthy controls and found that SWE showed higher stiffness in the CKD group (9.40 vs. 4.40 kPa, *p* = 0.002). In contrast, studies by Sayed et al. [21] and Shaker et al. [22] found increased stiffness with disease, suggesting that, in specific populations—especially children or those with diabetes-related pathology—tissue fibrosis and perfusion changes may yield higher SWE values.

This study demonstrated that SWE measurements of the right kidney showed a positive correlation with estimated glomerular filtration rate (eGFR).

In harmony with our findings, Bob et al. [23] reported that shear wave speed significantly decreased with worsening renal function. Specifically, patients with eGFR > 90 mL/min/1.73 m² had higher shear wave speeds (2.32 ± 0.83 m/s) compared to those in CKD stage 4 (1.62 ± 0.75 m/s, *p* = 0.03) and stage 5 (1.66 ± 0.72 m/s, *p* = 0.04). A cutoff value of ≤ 2.26 m/s predicted eGFR < 30 mL/min with 86.7% sensitivity and 95.6% negative predictive value (AUC = 0.692, *p* = 0.008). These findings confirm that renal stiffness decreases in parallel with functional deterioration. In accordance, Farris et al. [24] evaluated the relationship between interstitial fibrosis and renal function using various morphometric and visual techniques in renal biopsies from native and transplant kidneys. They reported strong correlations between fibrosis extent (as measured by Collagen III morphometry, Sirius Red, and visual trichrome scoring) and eGFR, with R² values ranging from 0.38 to 0.50 (*p* < 0.01–0.001).

SWE measures kidney tissue stiffness by analysing how shear waves propagate through it. Stiffer tissue indicates fibrosis or scarring, meaning lower kidney function. Contrasting our results, a study by Hassan et al. [25] found that cortical stiffness was inversely correlated with the eGFR (*r* = − 0.65, *P* < 0.001). Also, Leong et al. [26] reported a negative correlation between SWE values and eGFR (*r* = − 0.576, *P* < 0.0001). Furthermore, Arndt et al. found that stiffness showed inverse correlation with eGFR (*r* = -0.47, *P* = 0.0003). These discrepancies may be attributed to differences in population, sample sizes, pathological conditions, and methodologies.

In the present study, SWE values of the right kidney predicted eGFR < 30 mL/min/1.73 m² with reasonable accuracy. The ROC analysis revealed an AUC of 0.706, and at a cutoff value of 6.9 kPa, SWE had 71% sensitivity and 79% specificity. These findings underscore SWE’s potential as a reliable screening tool for identifying patients at risk of advanced renal impairment, particularly when biopsy is contraindicated.

In accordance, Rashied et al. [27] evaluated the role of SWE in assessing renal stiffness and found that SWE showed excellent diagnostic accuracy with an AUC of 0.932 and a cutoff of > 5.1 kPa, yielding 82.14% sensitivity and 100% specificity. These results parallel ours in supporting the diagnostic utility of SWE in detecting early renal parenchymal changes. Also, Qiang et al. [28] prospectively evaluated 103 critically ill patients using SWE to assess its diagnostic performance in detecting acute kidney injury (AKI). They found significantly *increased* stiffness values in AKI patients, with optimal cutoffs of 9.9 kPa (upper pole medulla), 2.9 kPa (middle cortex), and 4.4 kPa (middle medulla), yielding AUCs of 0.737–0.784. These results support SWE’s role as a non-invasive diagnostic tool for acute renal pathology. In addition, Turgutalp et al. [11] assessed the utility of SWE in evaluating renal stiffness. They found that SWE-derived Young’s modulus (YM) was highly sensitive and specific for diagnosing interstitial fibrosis (IF), with a sensitivity of 89%, specificity of 90%, and a positive predictive value (PPV) of 91% at a threshold of > 15 kPa.

In the current study, non-LN patients showed a significant negative correlation between right kidney SWE values and interstitial fibrosis percentage, suggesting that increased histological fibrosis is associated with reduced renal tissue elasticity.

In contrast, Leong et al. [29] found that the mean YM increased as the percentage of interstitial fibrosis and tubular atrophy increased. In addition, a systematic review and meta-analysis by Filipov et al. [30] reported a moderate positive correlation between SWE-derived stiffness and fibrosis severity, with pooled Pearson’s *r* = 0.48 (CI: 0.20–0.69) and Spearman’s *r* = 0.57 (CI: 0.35–0.72).

In the present study, patients with lower eGFR levels tended to be older and more hypertensive, with significantly lower SWE values. This aligns with known risk factors for CKD progression and supports integrating SWE into comprehensive clinical assessment, particularly for early identification of patients with high-risk profiles.

eGFR naturally declines with age, even in the absence of kidney disease. Therefore, older individuals are more likely to have lower eGFR values simply because of aging [31]. GFR declines, perhaps inexorably, with normal ageing, usually beginning after 30–40 years of age. The rate of decline may accelerate after age 50–60 years [32]. Lower eGFR indicates reduced kidney function, and this decline is strongly associated with an increased risk of developing hypertension and experiencing its complications [33]. In accordance, Li et al. [34] found that compared to those with normal or near-normal eGFR, subjects with lower levels had more elders and a higher proportion of hypertension (HBP).

In the current study, LN patients had significantly higher eGFR and lower creatinine levels than those with CKD/ESRD.

In accordance, Chrysostomou et al. [35] analyzed data from the Swedish Renal Registry (2006–2021) to compare long-term outcomes in patients with lupus nephritis–associated CKD (LN-CKD) to those with primary glomerular diseases (PGD) and other CKD etiologies (mainly diabetic nephropathy and nephroangiosclerosis). Their cohort included 317 LN patients with better kidney function than those in the Other-CKD group. Also, Farinha et al. [36] compared clinical presentations between proliferative and membranous forms of LN and found that membranous LN patients exhibited significantly lower serum creatinine levels compared to proliferative LN patients, and that eGFR ≤ 75 mL/min/1.73 m² at one year post-treatment was the strongest predictor of CKD progression (HR 23, 95% CI: 8–62, *p* < 0.001).

Importantly, the majority of CKD cases are not histologically characterized because kidney biopsy is not routinely performed in common etiologies such as diabetic nephropathy and hypertensive kidney disease. As a result, many patients lack tissue-based assessment of chronicity or fibrosis. This underscores the need for complementary non-invasive techniques, such as SWE, which can provide additional structural information in patients who would otherwise not undergo biopsy.

This study is limited by its small sample size, single-center design, and cross-sectional nature, which may affect the generalizability of the findings. Inter-operator variability and technical factors inherent to SWE could also influence measurements. Shear wave elastography of the kidney is technically challenging due to the intrinsic anisotropy of renal tissue, which affects shear-wave propagation and may introduce measurement variability. Another important limitation is the relatively high BMI of the study population. Increased adipose tissue can attenuate ultrasound waves and reduce SWE signal penetration, thereby affecting the accuracy and reproducibility of stiffness measurements. Larger multicenter studies with standardized measurement protocols are needed to validate SWE’s diagnostic role across diverse renal pathologies.

## Conclusions

Supersonic shear wave elastography (SWE) is a promising noninvasive imaging modality for evaluating renal parenchymal abnormalities. In this study, renal stiffness measurements correlated with kidney function and histopathological changes, particularly interstitial fibrosis, supporting its potential role as an adjunct to biopsy in the assessment of chronic kidney disease. Larger prospective studies are warranted to confirm its diagnostic accuracy, standardize protocols, and define its role in clinical decision-making.

## Supplementary Information

Below is the link to the electronic supplementary material.

Supplementary Material 1

## Data Availability

The datasets generated and/or analysed during the current study are not publicly available due to privacy, ethical, and consent restrictions but are available from the corresponding author on reasonable request and with appropriate institutional approvals.

## Abbreviations

ANA: Antinuclear Antibody
anti-dsDNA: Anti–Double Stranded DNA
AUC: Area Under the Curve
CKD: Chronic Kidney Disease
CKD/ESKD: Chronic Kidney Disease / End-Stage Kidney Disease
ESKD: End-Stage Kidney Disease
eGFR: Estimated Glomerular Filtration Rate
GE: General Electric
HCV: Hepatitis C Virus
IF: Interstitial Fibrosis
IRB: Institutional Review Board
IQR: Interquartile Range
ISN/RPS: International Society of Nephrology / Renal Pathology Society
LN: Lupus Nephritis
MPGN: Membranoproliferative Glomerulonephritis
MRI: Magnetic Resonance Imaging
ROC: Receiver Operating Characteristic
SSI: Supersonic Shear Imaging
SWE: Shear Wave Elastography
TMA: Thrombotic Microangiopathy
YM: Young’s Modulus
C3: Complement Component 3
C4: Complement Component 4
B-mode: Brightness Mode Ultrasound
SD: Standard Deviation
PPV: Positive Predictive Value
HBP: High Blood Pressure
AKI: Acute Kidney Injury
