# Supplementary material for: Shear wave elastography as a reliable tool in the prediction of renal histopathological abnormalities
Source: BMC Med Imaging. 2026 Jan 24;26:55. doi: 10.1186/s12880-025-02137-7 (PMC12849660; doi:10.1186/s12880-025-02137-7)
Supplement: Supplementary file 1 — Supplementary Material 1 [file 12880_2025_2137_MOESM1_ESM.docx]

**Supplementary files**

| **Supplementary Table 1. Histopathological data of LN patients (n=20)** | | | | |
| --- | --- | --- | --- | --- |
| **No. of glomeruli** *(Median – IQR)* | | | 13.5 (10-24) | |
| **Class** | **Class I**  **Class II**  **Class III**  **Class IV**  **Class V**  **Class VI** | | 1 (5)  1 (5)  0  12 (60)  3 (15)  3 (15) | |
| **Activity indices (Class IV) (n=12)** | | | | |
| **Activity parameter** | **% of active lesion** | | | **Score** |
| **Endocapillary hypercellularity** | 54.5 (41-79.75) | | | 2.5 (2-3) |
| **Hyaline lesions** | 27 (3.5-49.25) | | | 1.5 (025-2) |
| **Cellular crescents** | 19.5 (9.13-37) | | | 2 (2-3.5) |
| **Fibrinoid necrosis** | 0 (0-6) | | | 0 (0-0.75) |
| **Neutrophils infiltration/karyorrhexis** | 21 (5.5-31) | | | 1 (1-2) |
| **Interstitial infiltration**  **Negative**  **0-25%**  **25-50%**  **> 50%** | 1 (8.3)  7 (58.3)  4 (33.3)  0 | | | 1 (1-1) |
| **Activity Index** *(Median – IQR)* | | 8 (7-10.75) | | |
| **Chronicity indices (Class IV) (n=12)** | | | | |
| **Chronicity parameter** | **% of chronic lesion** | | **Score** | |
| **Sclerotic glomeruli** | 20 (9.5-44.25) | | 1 (1-2) | |
| **Fibrous crescents** | 6.5 (0-17.75) | | 1 (0-1) | |
| **Atrophic tubules**  **Negative**  **0-25%**  **25-50%**  **> 50%** | 2 (16.7)  5 (41.7)  3 (25)  2 (16.7) | | 1 (1-2) | |
| **Interstitial fibrosis**  **Negative**  **0-25%**  **25-50%**  **> 50%** | 2 (16.7)  5 (41.7)  3 (25)  2 (16.7) | | 1 (1-2) | |
| **Chronicity Index** *(Median – IQR)* | | 4 (3-6) | | |

| **Supplementary Table 2: Correlations of Shear Wave Speed of both kidneys with the studied variables (LN cases: n=20)** | | | | |
| --- | --- | --- | --- | --- |
| Parameter | Right kidney | | Left kidney | |
|  | r | p | r | p |
| Age | -0.16 | 0.49 | -0.231 | 0.32 |
| Creatinine | -0.04 | 0.84 | -0.08 | 0.72 |
| eGFR | 0.06 | 0.8 | 0.157 | 0.51 |
| Protein | 0 | 1 | -0.267 | 0.26 |
| **Endocapillary hypercellularity score** | -0.15 | 0.58 | -0.45 | 0.1 |
| **Hyaline lesions score** | -0.05 | 0.85 | -0.187 | 0.52 |
| **Cellular crescents score** | -0.32 | 0.26 | 0.136 | 0.64 |
| **Fibrinoid necrosis score** | -0.02 | 0.94 | 0.3 | 0.28 |
| **Neutrophils infiltration/karyorrhexis score** | 0.06 | 0.83 | 0.86 | 0.007* |
| **Interstitial infiltration score** | 0.175 | 0.54 | -0.03 | 0.91 |
| **Activity index** | -0.125 | 0.68 | 0.33 | 0.25 |
| **Sclerotic glomeruli score** | 0.05 | 0.85 | 0.02 | 0.92 |
| **Fibrous crescents score** | 0.038 | 0.89 | 0.38 | 0.17 |
| **Tubular atrophy score** | 0.342 | 0.21 | -0.04 | 0.88 |
| Interstitial fibrosis score | 0.342 | 0.21 | -0.04 | 0.88 |
| Chronicity index | 0.428 | 0.12 | -0.002 | 0.99 |

| **Table 3: Comparison of Patient Characteristics and Shear Wave Speed in patients with and without LN** | | | |
| --- | --- | --- | --- |
|  | LN (n=20) | Non-LN (n=31) | P |
| Age | 31.05±8.59 | 36±13.09 | 0.14 |
| Gender:  Male: n(%)  Female: n(%) | 3 (15)  15 (85) | 18 (58.1)  13 (41.9) | 0.003^a^ |
| DM: n(%) | 0 | 6 (19.4) | 0.07 ^a^ |
| HTN: n(%) | 5 (25) | 16 (51.6) | 0.059^b^ |
| Serum creatinine | 1.25 (0.9-4.37)^b^ | 2.5 (1.4-9.3) | 0.053 |
| eGFR | 63.6 (13.4-89.7)^b^ | 26.3 (6.9-65.1) | 0.09 |
| 24 hours urinary protein | 2400 (1990-3700) | 2600 (1450-5000) | 0.78 |
| Shear wave speed:  Right kidney:  Left kidney: | 6.9 (4.97-8.65)  8.65 (6.92-10.45) | 7 (5.6-8.1)  7.5 (6.6-9.1) | 1  0.21 |
